# Inhibin-A and Decorin Secreted by Human Adult Renal Stem/Progenitor Cells Through the TLR2 Engagement Induce Renal Tubular Cell Regeneration

**DOI:** 10.1038/s41598-017-08474-0

**Published:** 2017-08-15

**Authors:** Fabio Sallustio, Claudia Curci, Alessandra Aloisi, Chiara Cristina Toma, Elisabetta Marulli, Grazia Serino, Sharon Natasha Cox, Giuseppe De Palma, Alessandra Stasi, Chiara Divella, Rosaria Rinaldi, Francesco Paolo Schena

**Affiliations:** 1 University of Bari, Department of Emergency and Organ Transplantation, Piazza G. Cesare 11, 70124 Bari, Italy; 2 C.A.R.S.O. Consortium, Strada Prov. le Valenzano-Casamassima Km 3, 70100 Valenzano (Ba), Italy; 3 Schena Foundation, Strada Prov. le Valenzano-Casamassima Km 3, 70100 Valenzano (Ba), Italy; 4 Consiglio Nazionale delle Ricerche (CNR), Institute of Nanoscience, Via Arnesano 16, 73100 Lecce, Italy; 5 Institute of Microelectronics and Microsystems (C.N.R.- I.M.M.), via Monteroni, Campus Ecotekne, 73100 Lecce, Italy; 6 University of Salento, Mathematics and Physics “E. De Giorgi” Department, University of Salento, 73100 Lecce, Italy; 7 National Institute of Gastroenterology “S. de Bellis”, Research Hospital, Castellana Grotte, Bari, 70013 Italy

## Abstract

Acute kidney injury (AKI) is a public health problem worldwide. Several therapeutic strategies have been made to accelerate recovery and improve renal survival. Recent studies have shown that human adult renal progenitor cells (ARPCs) participate in kidney repair processes, and may be used as a possible treatment to promote regeneration in acute kidney injury. Here, we show that human tubular ARPCs (tARPCs) protect physically injured or chemically damaged renal proximal tubular epithelial cells (RPTECs) by preventing cisplatin-induced apoptosis and enhancing proliferation of survived cells. tARPCs without toll-like receptor 2 (TLR2) expression or TLR2 blocking completely abrogated this regenerative effect. Only tARPCs, and not glomerular ARPCs, were able to induce tubular cell regeneration process and it occurred only after damage detection. Moreover, we have found that ARPCs secreted inhibin-A and decorin following the RPTEC damage and that these secreted factors were directly involved in cell regeneration process. Polysaccharide synthetic vesicles containing these molecules were constructed and co-cultured with cisplatin damaged RPTECs. These synthetic vesicles were not only incorporated into the cells, but they were also able to induce a substantial increase in cell number and viability. The findings of this study increase the knowledge of renal repair processes and may be the first step in the development of new specific therapeutic strategies for renal repair.

## Introduction

Acute kidney injury (AKI) is characterized by quick deterioration of the kidney function and this event is increasing in the last years^1, 2^.

Most cases of AKI arise from renal ischemia, drug toxicity or metal exposure. Cisplatin is a widely used cancer chemotherapeutic agent that gives renal damage. It is used to treat various types of cancers, including sarcomas, some carcinomas (e.g. small cell lung cancer and ovarian cancer), lymphomas, and germ cell tumors. Despite the newly developed targeted therapies in oncologic treatment, cisplatin is still in use and nephrotoxicity remains a major concern. Dose-related and cumulative renal insufficiency, including AKI, is the major dose-limiting toxicity of cisplatin^3, 4^. Several pharmacologic therapies that accelerate recovery and improve survival have been attempted. They were efficacious in experimental models but failed to manifest any substantial beneficial effect in the clinical practice^5^. This suggests that the development of more successful therapies requires a different approach.

Resident human adult renal CD133^+^/CD24^+^ progenitor cells (ARPCs) can participate in renal repair processes and might therefore be considered a good candidate for a future therapy to improve regeneration in AKI^6, 7^. Nevertheless, recent studies indicate that the predominant mechanism of repair after ischemic renal tubular injury is the regeneration by surviving tubular epithelial cells^8^, suggesting that ARPCs could contribute to renal regeneration by means of paracrine/endocrine mechanisms. These cells have a multipotent differentiation ability, including the capacity to differentiate in tubular epithelial cells, osteogenic cells and adipocytes^9–11^.

CD133^+^/CD24^+^ renal progenitor cells are present at glomerular and tubular levels in normal kidneys, they express the toll-like receptor-2 (TLR2) that may function as damage sensor and activate damage recovering mechanisms^11^.

Recent cell-fate tracking studies suggest that the renal tubule repair process depends principally on the kidney epithelial cells that can lose their phenotype, plausibly dedifferentiating, and can adopt a stem cell fate expressing the CD133 and CD24 markers^12, 13^. Other similar studies showed that unipotent singly fated clones constantly maintain and self-preserve the renal mouse kidney tissue throughout life and have renal progenitor characteristics. After kidney damage, these precursors are activated by WNT signals and are able to regenerate new collective ducts or proximal tubules segments through the expansion of single clones^14^.

Anyway, all these studies agree that CD133^+^/CD24^+^ cells have high regenerative and reparative phenotype with an important role in the setting of renal damage repair.

Here we show that ARPCs can regenerate both physical and cisplatin-induced chemical damage through the secretion of regenerative molecules and microvesicles containing inhibin-A (Inhb-A) and decorin (DCN). Moreover, we demonstrate that this process is mediated by TLR2 that is constitutionally expressed on the ARPCs and that the secreted chemokines could be clinically useful in promoting the reparative process of human renal proximal tubular epithelial cells (RPTECs).

## Results

### The tubular ARPCs can repair physically injured or chemically damaged RPTECs

ARPCs were isolated and characterized as previously described^11, 15–17^ and we confirmed that they showed a positive staining for the following markers:CD133, CD24, PAX2, BMI-1, Oct-4 and CD44.

We investigated whether the ARPCs were able to restore a physical damage induced on RPTECs using wound-healing scratch assay that mimics *in vivo* cell migration^18^.

**Figure 1:**
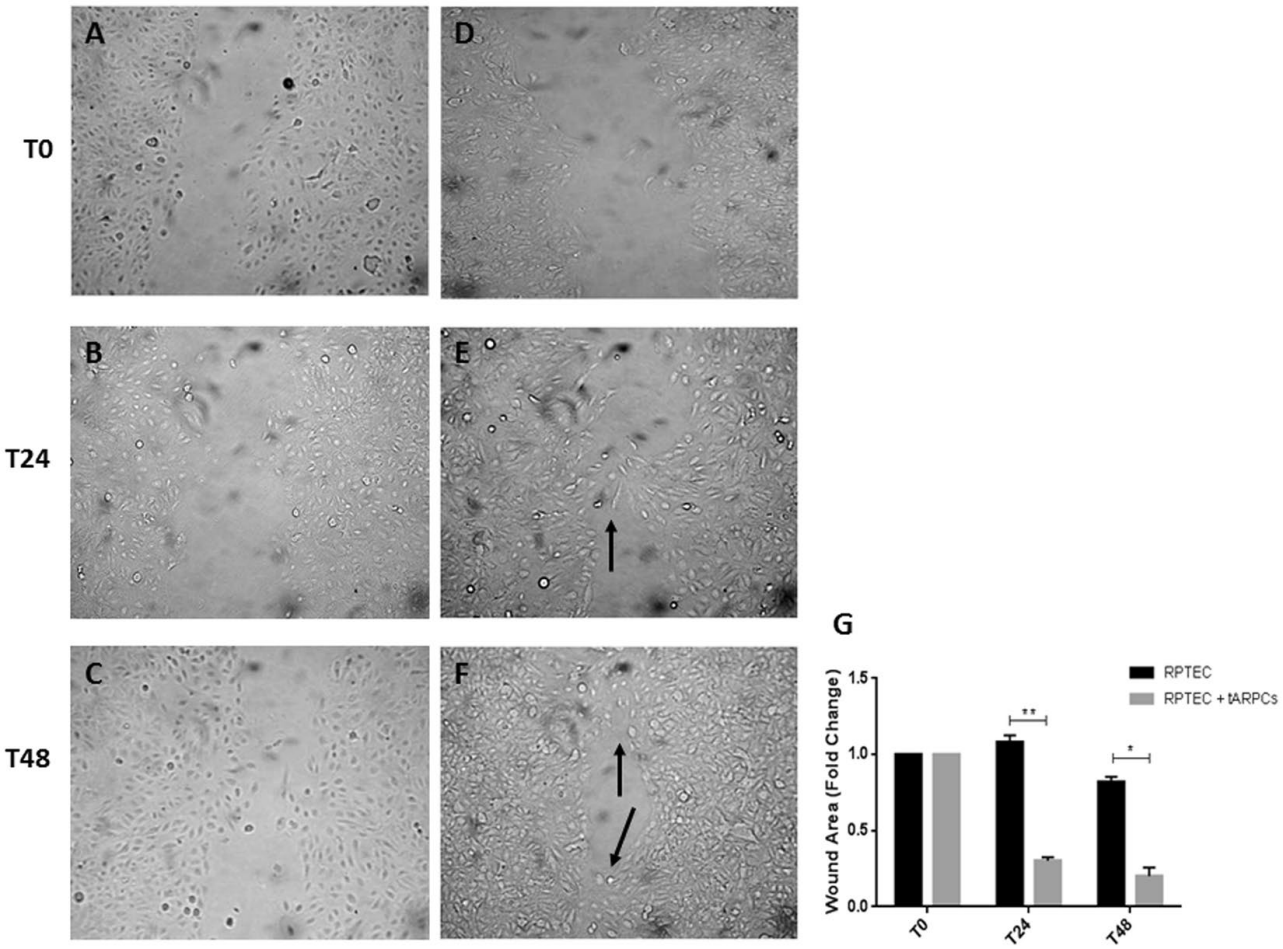
tARPCs can repair RPTECs physically injured. The wound healing assay showed that tARPCs can repair physically damaged RPTECs. A scratch was performed on tubular cell monolayers to simulate a physical damage. Subsequently, tubular cells were incubated alone (**A**–**C**) or in co-culture with ARPCs on transwells (**D**–**F**). Images were captured at phase contrast microscopy at intervals of 0, 24 and 48 hours (T0, T24 and T48, respectively) to detect the gradual repair of the gap. When RPTECs were in co-culture with tARPCs, they displayed an increased capacity to fill in the damaged area (**E**,**F)**, when compared to RPTECs cultured alone (**B**–**C**). After 24 hours, the scratch in co-cultured RPTECs already started to close and contact points were seen (E, black arrow). At 48 hours, many more junction points were observed between gaps in RPTECs co-cultured with ARPCs (**F**, black arrows) compared RPTEC cultured alone. (**G**) Quantization results are expressed as a ratio between area of scratches at T48 and at T24 compared to the scratch area at T0. Plots represent 3 independent experiments using ARPCs from 3 different subjects; *P < 0.05, **P < 0.005.

RPTECs were mechanically displaced by scratching a line through the cell layer and the gap was visually inspected (Fig. 1, T24 and T48, respectively) during the cell migration process to fill in the damaged area. When RPTECs were in co-culture with tARPCs, they displayed an increased ability in filling in the damaged area (T48 panel), when compared to RPTEC cultured alone. After 24 hours, the scratch in co-cultured RPTECs already started to close (Fig. 1, T24). At 48 hours, many more junction points were observed between gaps in RPTECs-ARPCs co-cultures (Fig. 1, T48) compared RPTEC cultured alone. Quantization results showed that tARPCs induced a significant reduction of scratch gaps (Fig. 1G).

Then, we investigated whether ARPCs were able to regenerate damaged RPTECs treated with cisplatin, an antineoplastic drug that causes cell damage, inhibition of cell proliferation and apoptosis.

To study the interactions occurring between ARPCs and injured RPTECs, we set up an *in vitro* model of cisplatin-induced cell toxicity. RPTECs were treated with 2.5 µmol/l cisplatin for 6 hours and, after drug removal, cell proliferation assays were performed.

ARPCs ability to regenerate cisplatin-damaged tubular cells was investigated using a co-culture system in which cells were physically separated by a transwell that only allowed the transition of secreted molecules.

**Figure 2:**
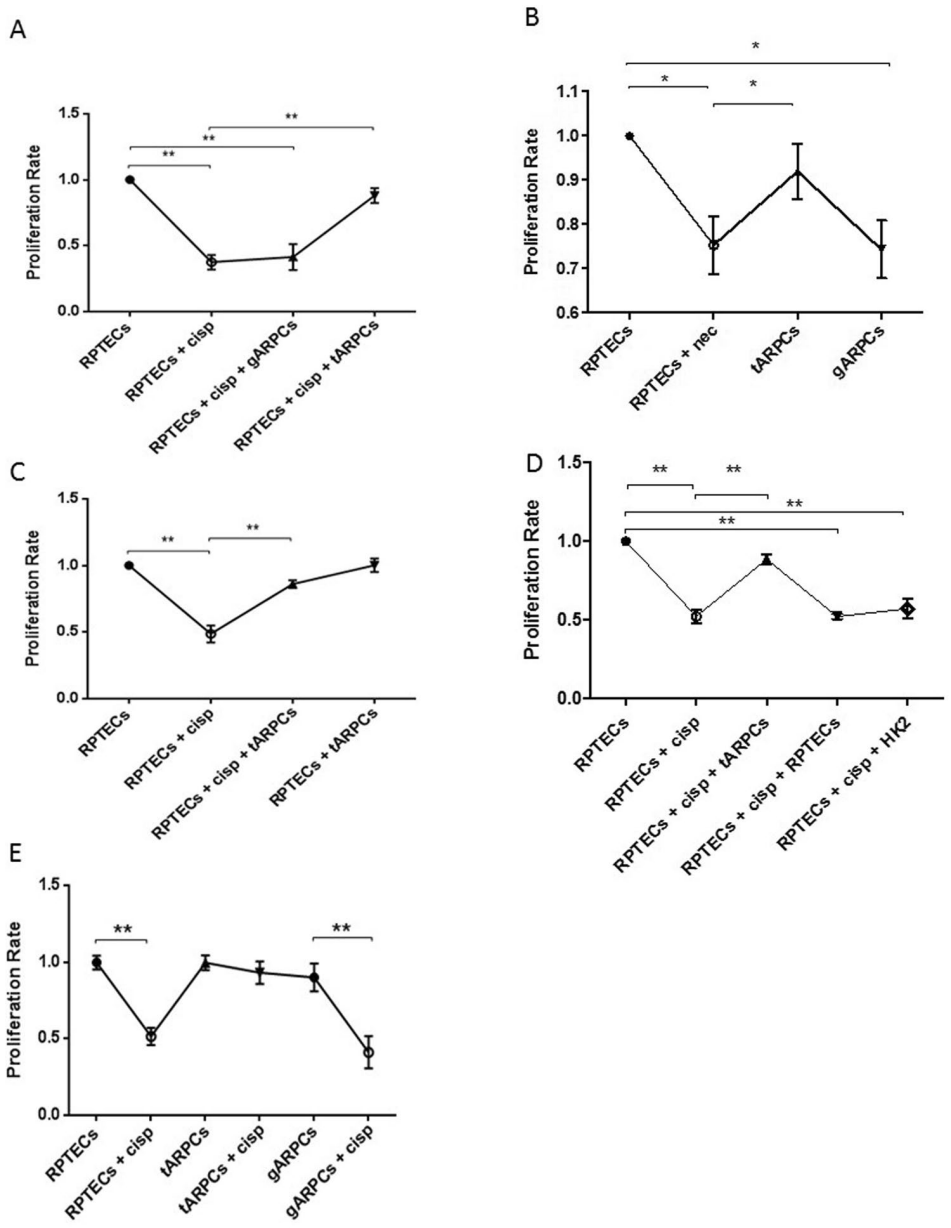
tARPCs can repair cisplatin damaged RPTECs and necrotic cells. RPTEC BrdU proliferation assays showed the capacity of ARPCs to induce the regeneration of cisplatin-damaged tubular cells. (**A**) RPTEC proliferation rate at 4 days after cisplatin treatment (2.5 µmol/l) significantly decreased compared with healthy cells. When damaged cells were co-cultured with tARPCs, they recovered their proliferation rate. The gARPCs did not influence RPTEC proliferation rate. (**B**) Necrosis was induced on RPTECs and the cells were cultured with or without ARPCs for 4 days after inducing damage. In ARPCs absence RPTECs did not recover their proliferation rate and was significantly lower compared to non-damaged cells. RPTEC proliferation rate recovered only when damaged cells were co-cultured with tARPCs, whereas with gARPCs the recovery was absent (**C**). tARPCs induced the repair process only after damage perception. The proliferation rate of healthy RPTECs did not change when they were co-cultured with tARPCs without cisplatin (RPTECs + tARPCs) for 4 days. (**D**) The repair process was induced specifically by tARPCs. No significant increase in cell proliferation was observed when damaged RPTECs were co-cultured with healthy HK2 cells or RPTECs for 4 days. (**E**) Cell culture experiments showed that RPTECs and gARPCs, but not tARPCs, decreased their proliferation rate after cisplatin exposition (2.5 mmol/l drug for 6 h). Plots represent 5 independent experiments using ARPCs from 5 different subjects; *P < 0.05, **P < 0.005.

After 4 days cisplatin treatment, RPTECs cell proliferation rate significantly decreased compared with non-damaged RPTECs while damaged-RPTECs co-cultured with tARPCs proliferated like untreated cells. Instead, the co-culture experiment with glomerular ARPCs (gARPCs) was not able to restore damaged RPTEC cell proliferation (Fig. 2A).

Similarly, the tARPCs, repaired necrotic RPTECs induced by 6 mM deoxyglucose and 10 mM sodium azide^19^ treatment for 12 h. Damaged cells were then cultured with or without tARPCs or gARPCs for 4 days. RPTEC proliferation rate dropped in the absence of ARPC co-culture compared with healthy cells. RPTEC recovery occurred in the co-culture with tARPCs, whereas it was absent in the co-culture with gARPCs (Fig. 2B).

### The repair process is specifically induced by tARPCs only after damage perception

ARPCs progenitor cells can induce proliferation only in response to damage, as cell proliferation was not significantly different when healthy RPTECs were co-cultured for 4 days with tARPCs (Fig. 2C). Then, we checked whether the repair process was a specific prerogative of tARPCs. We co-cultured damaged RPTECs together with healthy immortalized tubular cells HK2 or RPTECs for 4 days and we did not observe a significant enhancement of the cell proliferation rate. These results showed that RPTECs recovery was induced exclusively by tARPCs and not by HK2 cells or RPTECs (Fig. 2D). Moreover, we checked the resistance of both tARPCs and gARPCs and RPTECs to cisplatin treatment (2.5 mmol/l drug for 6 h). We found that the RPTECs and the gARPCs were very susceptible to cisplatin, whereas the tARPCs were more resistant (Fig. 2E). This could explain why the reparative effect is given by tARPCs and not by gARPCs.

### The tARPCs can abrogate cisplatin-induced apoptosis of RPTECs

**Figure 3:**
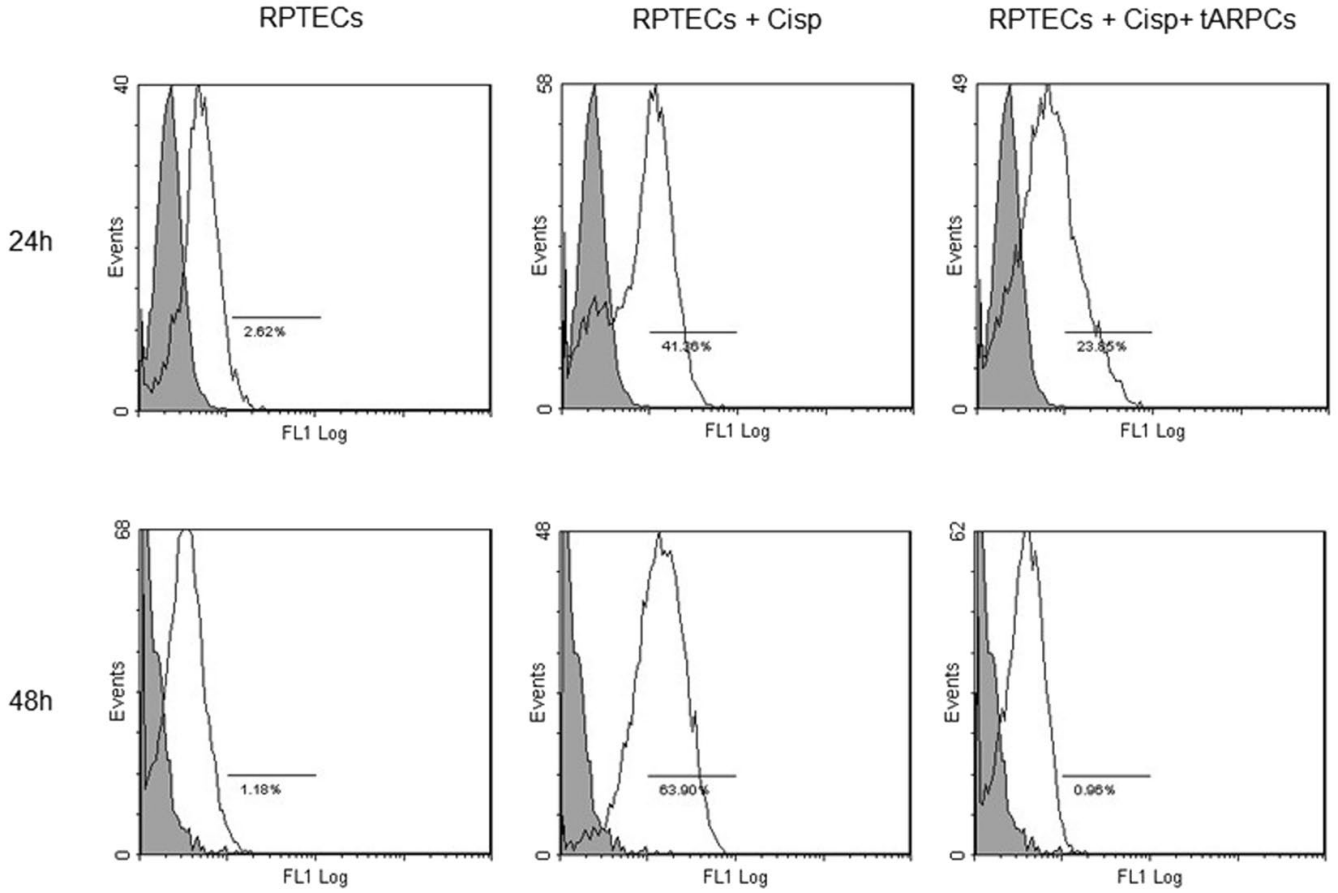
tARPCs can abrogate cisplatin-induced apoptosis of RPTECs. After cisplatin administration, the number of cleaved-caspase 3 positive RPTECs significantly increased to 41% after 24 hours and to and 63.9% after 48 hours. Conversely, when RPTECs were co-cultured with tARPCs, apoptotic cells decreased to 24% after 24 hours, whereas after 48 hours, apoptosis was completely blocked and RPTECs did no longer express cleaved-caspase 3. Results are representative of three independent experiments.

RPTECs regeneration after cisplatin injury not only depends on the enhanced proliferation rate, but could also depend on other mechanisms i.e. suppression of apoptosis. We tested this hypothesis by setting up a model of cisplatin-induced cell toxicity and evaluated the cytofluorimetric expression of cleaved-caspase 3. After cisplatin administration, the number of cleaved-caspase 3 positive RPTECs significantly increased to 41% after 24 hours and to and 63.9% after 48 hours. Conversely, when RPTECs were co-cultured with tARPCs, apoptotic cells decreased to 24% after 24 hours, whereas after 48 hours, apoptosis was completely blocked and RPTECs did no longer express cleaved-caspase 3 (Fig. 3 and Supplementary Figure 1).

### The repair induced by tARPCs is mediated by the Toll-like receptor 2

**Figure 4:**
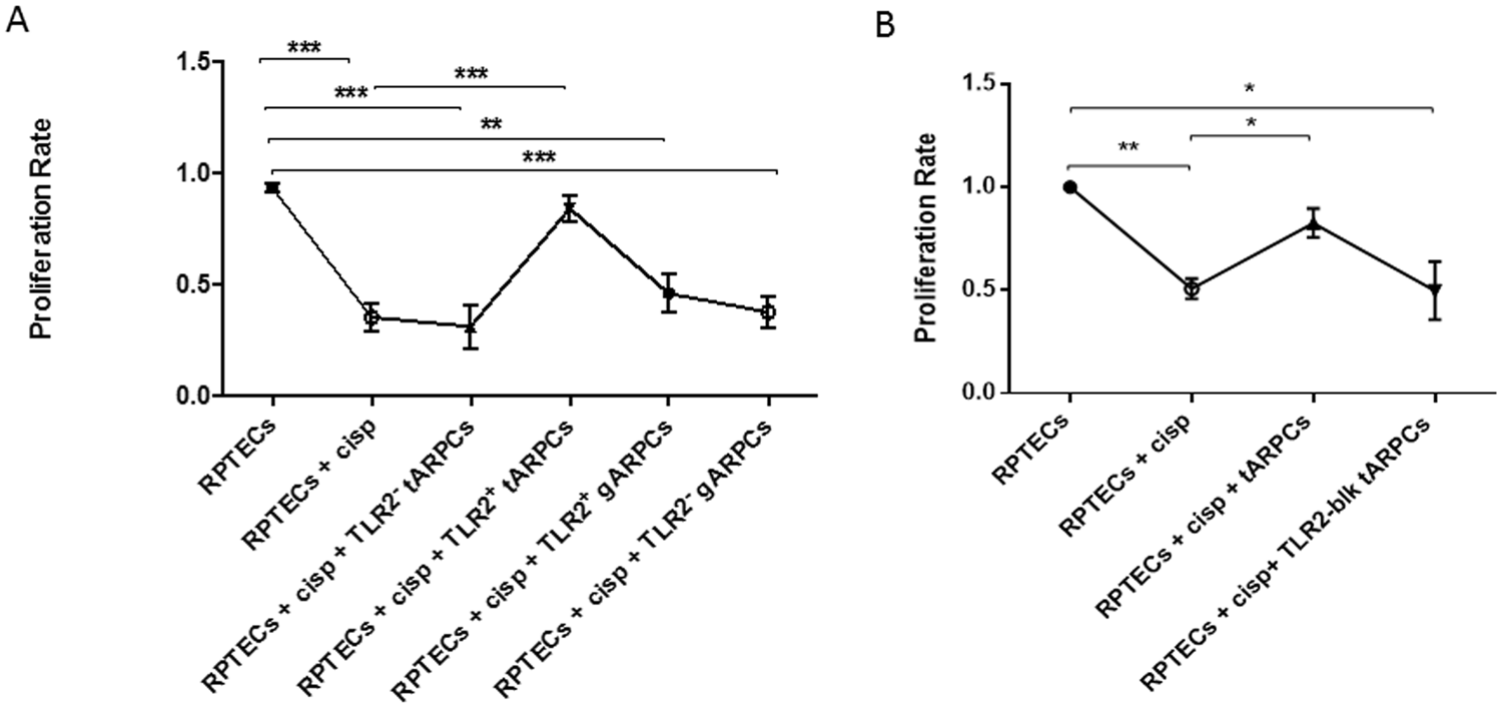
The repair induced by tARPCs was mediated by the Toll-like receptor-2. (**A**) BrdU cell proliferation assays showed that TLR2^+^ tARPCs were able to induce the repair of cisplatin damaged RPTECs in the co-culture system. On the contrary, the TLR2^−^ tARPCs and the TLR2^+^ gARPCs did not change the proliferation rate of damaged cells. (**B**) Cisplatin damaged RPTECs showed a limited proliferation rate in co-culture with tARPCs when TLR2 on renal progenitors was neutralized by a specific TLR2 blocking antibody. Plots represent 5 independent experiments using ARPCs from 5 different subjects; **P < 0.005, ***P < 0.0005.

**Figure 5:**
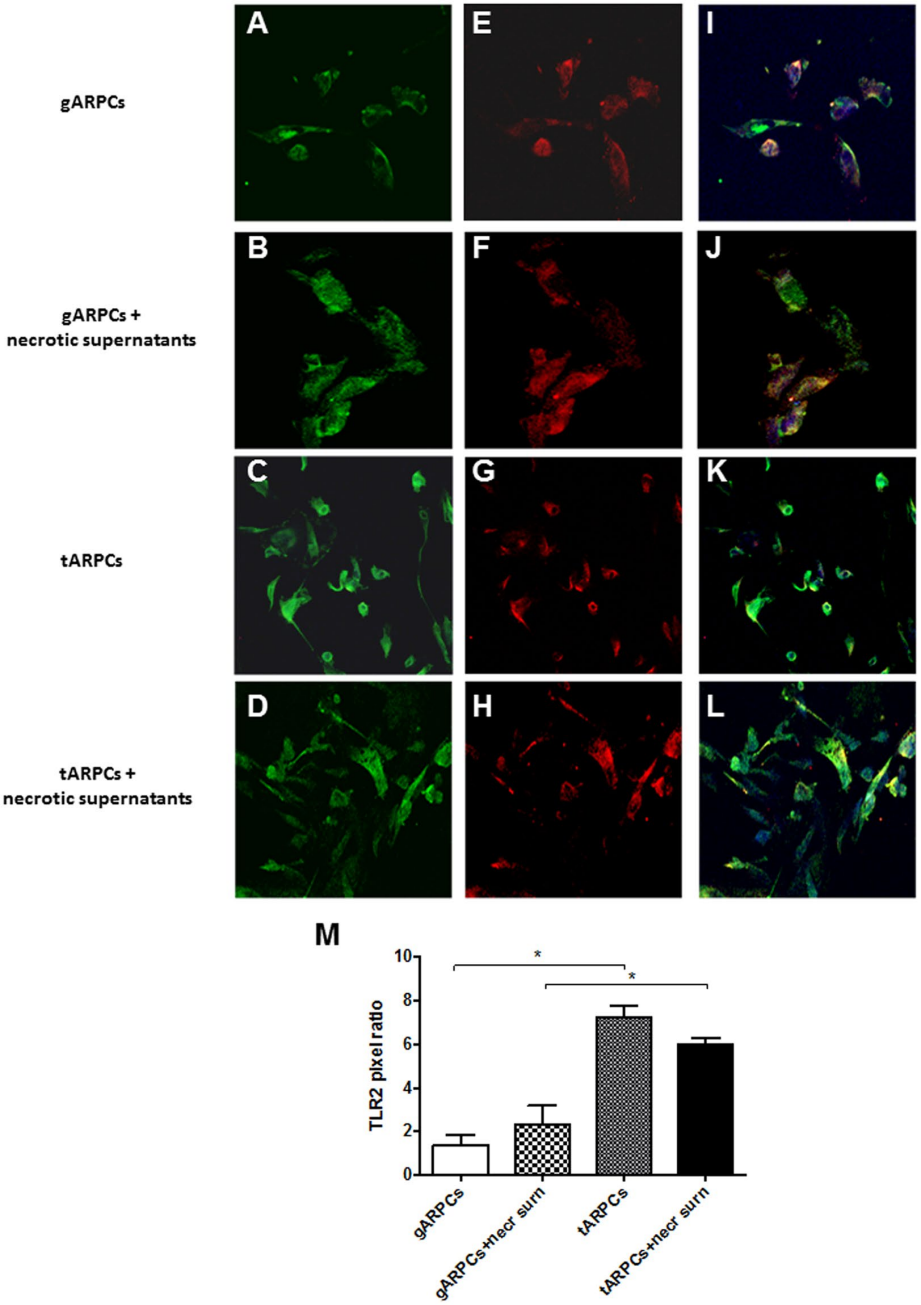
Difference of TLR2 expression in gARPCs and tARPCs following exposure to necrotic cell supernatants. Immunofluorescence experiments on ARPCs showing the TLR2 expression at basal conditions and after exposure to necrotic cell supernatants. (**A**–**D**) immunofluorescence showed the expression of TLR2 (green) in gARPCs, gARPCs + necrotic supernatant, tARPCs, and tARPCs + necrotic supernatans, respectively. (**E**–**H**) Immunofluorescence showed the expression of CD133 (red) in gARPCs, gARPCs + necrotic supernatant, tARPCs, and tARPCs + necrotic supernatants, respectively. (**I**–**L**) Double-label immunofluorescence showed the expression of TLR2 (green) and CD133 (red) in gARPCs, gARPCs + necrotic supernatant, tARPCs, and tARPCs + necrotic supernatant, respectively. Nuclei were stained with TO-PRO-3 (blue). Original magnification 40x. (**M**) Quantification of TLR2 expression by calculating the pixel ratio of positive cells in 10 different fields. TLR2 expression was significantly higher in tARPCs in basal condition and following exposure to necrotic cell supernatants. Reprinted from [Kidney International]^46^, Supplementary Figure S5, Copyright (2013), with permission from Elsevier.

We have previously shown that the TLR2 is overexpressed by ARPCs and that this receptor is able to activate progenitor cells^11^. Therefore, we investigated whether it is also involved in the repair processes mediated by tARPCs. We isolated TLR2^+^ and TLR2^−^ ARPCs through a magnetic labeling system and studied their ability to induce a functional response. In a co-culture system, only TLR2^+^ tARPCs were able to recover cisplatin damaged RPTECs. On the contrary, the TLR2^−^ renal progenitors did not give any functional effect (Fig. 4A). Moreover, neither TLR2^+^ gARPCs could repair cisplatin damaged RPTECs. The importance of this receptor in mediating RPTEC recovery was confirmed when we neutralized the receptor in co-culture experiments. We treated tARPCs with a specific TLR2-blocking antibody for 60 min and after several washes we used these cells for the co-culture. Cisplatin damaged RPTECs showed a limited proliferation rate when TLR2 on tARPCs was counteracted (Fig. 4B). The same results were obtained when tARPCs were silenced for the TLR2 and then co-cultured with cisplatin-damaged RPTECs (Supplementary Figure 2). Furthermore, we studied whether necrosis induction affected TLR2 expression in ARPCs in co-culture with the RPTECs. Immunofluorescence showed that the TLR2 expression did not change and that it remained higher in tARPCs compared to gARPCs (Fig. 5). All together these data show an essential role of the TLR2 in the damage repair process.

### ARPC microvesicles contain factors involved in RPTEC repair

**Figure 6:**
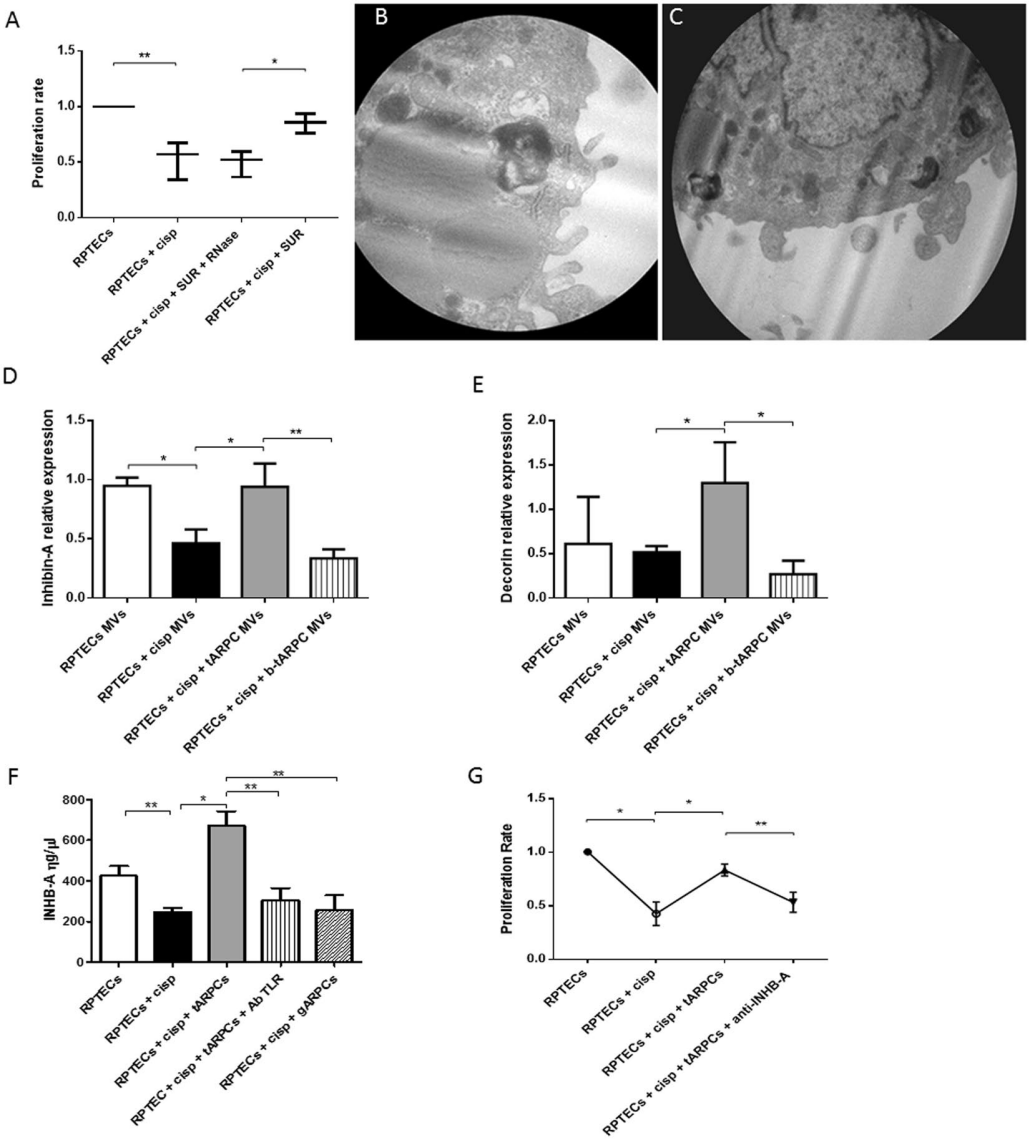
Inhibin-A and decorin were involved in the RPTEC repair. (**A**) Preconditioned supernatants from co-cultures induced an increase of RPTEC proliferation rate. Supernatants treated with 1 U/ml Rnase did not influence the proliferation rate. (**B**–**C**) Representative micrographs of transmission electron microscopy showing the release of MVs from the surface of a tARPC. Micrographs show the extrusion of MVs from the surface of the tARPC. Ultrathin sections, stained with led citrate were viewed by ZEISS EM910 electron microscope. Image acquisitions were performed with magnification of ×16000. (**D**) MVs isolated from the medium of regenerative condition (cisplatin-damaged RPTECs in co-culture with tARPCs), carried high levels of Inhb-A mRNA, these levels are comparable to the ones found in the supernatant of non-damaged RPTECs. Moreover, Inhb-A levels sharply decreased when TLR2 on tARPCs was blocked. (**E**) MVs isolated from the medium of the regenerative condition (cisplatin-damaged RPTECs in co-culture with tARPCs), carried high levels of Decorin mRNA, these levels were even higher than the ones detected in the supernatant of non-damaged RPTECs. Moreover, Decorin levels sharply decreased when TLR2 on tARPCs was blocked. (**F**) Inhb-A protein level increased in the regenerative condition (cisplatin-damaged RPTECs in co-culture with tARPCs), and this increase was abrogated when TLR2 receptor was blocked reaching levels similar to those obtained in the damaged condition. Inhb-A did not increased in the co-cultures of gARPCs with damaged-RPTECs. (**G**) BrdU cell proliferation assays showed that if we treated the regenerative medium (cisplatin-damaged RPTECs in co-culture with tARPCs) with the Inhb-A blocking antibody the damaged-RPTEC failed to recover their proliferation rate. Plots represent 5 independent experiments using tARPCs from 5 different subjects; *P < 0.05, **P < 0.005.

Since the tARPCs were physically separated from RPTECs, their reparative effect only could be mediated by paracrine factors. To confirm this assumption, we performed experiments with supernatants after 1 day of co-culture with cisplatin-damaged RPTECs (regenerative condition, cisplatin-damaged RPTECs in co-culture with tARPCs). After cisplatin damage, preconditioned supernatants induced an increase of RPTEC proliferation rate. If the supernatants were treated with 1 U/ml RNase, the regenerative effect was abolished (Fig. 6A). Furthermore, only supernatants from regenerative condition gave a functional affect whereas supernatants from tARPCs alone were not effective. These results suggested that the regenerative processes could be ascribed also to mRNA or miRNA shuttled by the microvesicles (MVs) secreted by tARPCs in addition to chemokines. We checked this with transmission electron microscopy after isolating MVs from tARPCs. Indeed, we found vesicular structures sized from 1 µm to 50 nm on tARPCs which originated directly from the budding of the plasma membrane (Fig. 6B).

### Inhibin-A and decorin are involved in the RPTEC repair

In our previous genome wide gene expression analysis, we found two upregulated chemokines in ARPCs compared to RPTECs^11^, the Inhibin-A (Inhb-A) and the decorin (DCN). They belong to the TGF-β signaling pathway and are involved in cell cycle regulation, increase of cell proliferation and inhibition of apoptosis^20–24^. We, therefore, checked the mRNA levels of these two chemokines in MVs isolated from tARPCs and RPTEC supernatants. We found that in MVs isolated from cisplatin-damaged RPTECs, Inhb-A mRNA significantly decreased, whereas MVs isolated from the medium of the regenerative condition carried levels of Inhb-A mRNA, comparable to the ones found in the supernatant of non-damaged RPTECs. Moreover, Inhb-A levels sharply decreased when TLR2 on tARPCs was blocked (Fig. 6D). In the same manner, MVs isolated from the regenerative condition carried high levels of Decorin mRNA and these levels were even higher than the ones detected in the supernatant of non-damaged RPTECs. Moreover, Decorin levels sharply decreased when TLR2 on tARPCs was blocked. (Fig. 6E). Interestingly, Inhb-A was also detected as free protein in the medium. The protein level increased in the medium of the regenerative condition, and this increase was abrogated when TLR2 receptor was blocked reaching levels similar to those detected in the damaged condition. (Fig. 6F). Inhb-A did not increased in the co-cultures of gARPCs with damaged-RPTECs. Moreover, when we treated the regenerative condition medium with the Inhb-A blocking antibody, the damaged-RPTEC failed to recover their proliferation rate, thus the reparative effect was abolished (Fig. 6G).

### Inhibin-A is expressed by damaged renal tubules enclosing tARPCs

**Figure 7:**
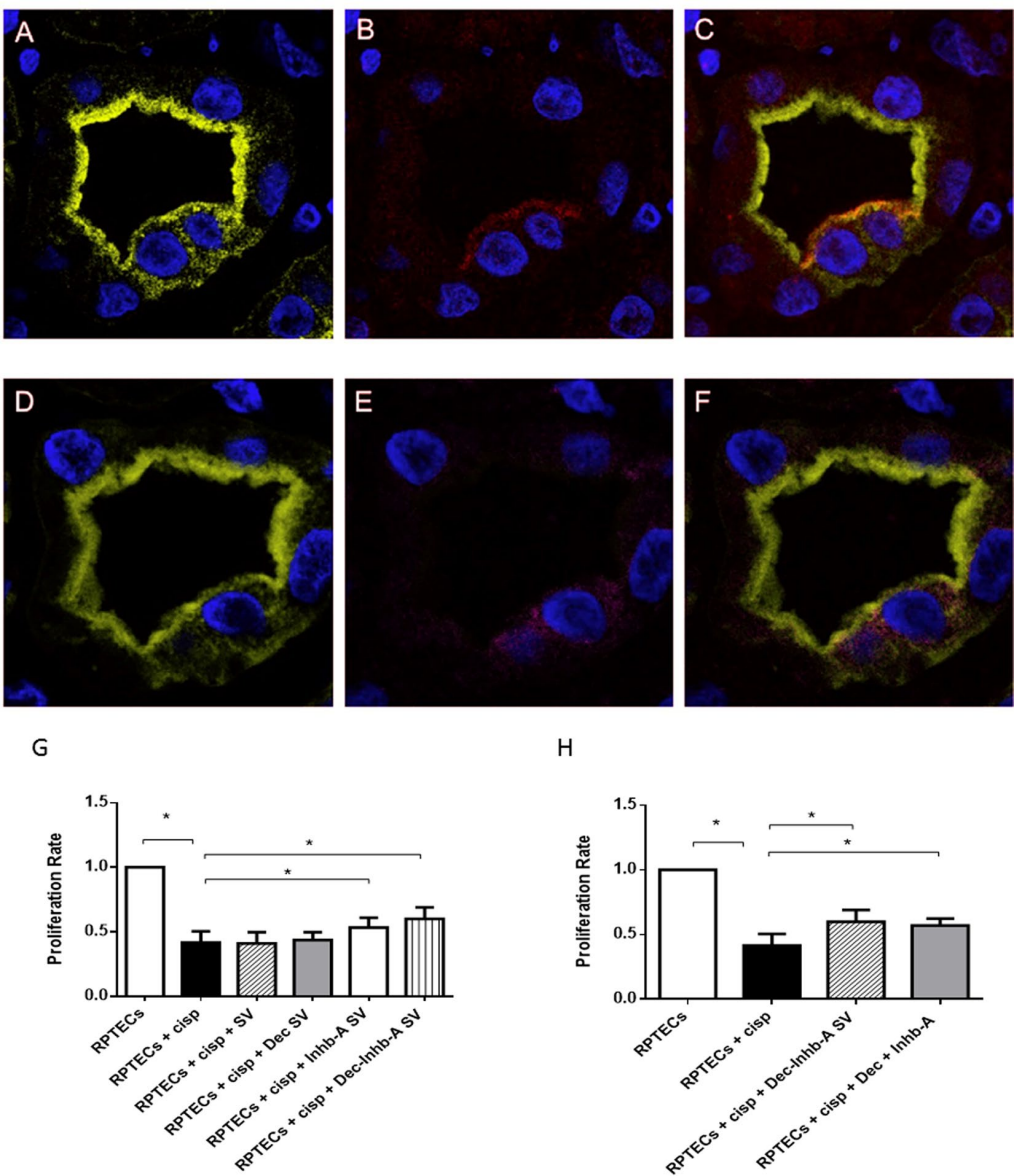
Inhibin-A is expressed by damaged renal tubules that enclosed tARPCs and its exogenous administration increased damaged-RPTEC proliferation rate. (**A**–**F**) Immunofluorescence on seriate tissue sections showed a proximal tubule labeled with Lotus (green, **A**,**D**) in which there are two CD133^+^progenitor cells (red, **B**) co-expressing Inhb-A (magenta, **E**). (**C**) Overlay of CD133 (red, **C**) and lotus (**A**). (**F**) Overlay of Inhb-A (magenta, (**E**) and lotus (**D**). The expression of Inhb-A colocalized with CD133^+^progenitor cells. To-pro-3 counterstained nuclei (blue). Original view: ×63. Reprinted from [Kidney International]^46^, Supplementary Figure S7, Copyright (2013), with permission from Elsevier. (**G**,**H**) BrdU proliferation assays showing that exogenous administration of Inhb-A and decorin increased damaged-RPTEC proliferation rate. (**G**) Inhb-A and decorin were loaded, individually or together, within polysaccharide synthetic vesicles (SV). The addition of Inhb-A and DCN SV to cisplatin-treated RPTECs led to a substantial increase in cell proliferation after 3 days of culture. DCN-SV alone were not able to increase the RPTEC proliferation rate, whereas INHB-A-SV gave a significant increase. On the other hand, the SV loaded with DCN and Inhb-A together gave a higher increase of cell proliferation. (**H**) BrdU proliferation assays showed that the exogenous administration of Inhb-A and decorin in the medium increased cell proliferation as proteins included in SV.

The tARPCs ability to secrete Inhb-A protein after tubular damage was confirmed with *ex vivo* immunofluorescence experiments. We used seriated tissue sections of renal biopsies from patients with delayed graft function (DGF) after transplant. The renal tubules of DGF patients showed that CD133^+^ cells, also had a positive staining for Inhb-A, whereas this expression was absent in transplanted patients without DGF (Fig. 7A–E).

### Exogenous administration of Inhb-A protein increases damaged-RPTEC proliferation rate

Inhb-A-loaded polysaccharide synthetic vesicles (Inhb-A-SV) and DCN-loaded polysaccharides synthetic vesicles (DCN-SV) were generated in a flow focusing microfluidic device. The natural polymer-based core-shell system was exploited for efficacious loading and delivery of functional molecules. We tested the cellular uptake and the INHB-A-SV and DCN-SV effectiveness. We found that these synthetic vesicles were incorporated in RPTECs and that Inhb-A and DCN SV in contact with cisplatin-treated RPTECs led to a substantial increase in cell number and viability after 3 days of culture. DCN-SV alone were not able to increase RPTEC proliferation rate, whereas INHB-A-SV gave a significant increase. Nevertheless, the SVs loaded with DCN and Inhb-A together gave a better functional response (Fig. 7G). However, one should consider that when we added Inhib-A and DCN proteins in the medium we had the same functional effect as for proteins included in SVs (Fig. 7H).

## Discussion

Recently, several cell-fate tracking studies have demonstrated that renal progenitor cells can directly or indirectly drive the renal repair process. In this setting, an important role could be ascribed to CD133^+^/CD24^+^ cells that are characterized by an enhanced regenerative potential with reparative properties^7, 12–14, 25^.

In this context, the understanding of ARPCs repair mechanisms could be very important. Here we have shown that tARPCs are able to drive RPTEC repair process induced by both chemical agents such as cisplatin, or by physical damage, such as a scratch in the epithelial layer. Moreover, we have found that tARPCs promoted the regeneration of tubular necrotic cells, but on the other hand, renal tubular damage cannot be repaired by the gARPCs, probably because these cells are more committed towards the glomerular phenotype. This is also in accordance with our data showing that tARPCs, but not gARPCs, were resistant to the cisplatin effect.

gARPCs are able to differentiate towards both a podocyte and tubular phenotype, while tARPCs already display a tubular committed phenotype^25^. Moreover, our previous microarray gene expression study showed that the transcriptional profile of tARPCs and gARPCs is very similar but a cluster of genes is able to differentiate tubular from glomerular progenitors^11^. Here, our results show a specific commitment of tARPCs in tubular repair and these results are in accordance with other studies that demonstrate the presence of a scattered tubular-committed population of progenitors that are highly resistant to death induced by toxic agents. In fact, in patients affected by acute tubular necrosis and in kidneys with chronic tubular damage, tARPCs are more than 20% while gARPCs are about 6%^25^ of all tubular cells. In addition, tARPCs are more resilient to injury compared to the surrounding proximal tubular cells^26^ and after kidney damage, these precursors are able to regenerate new collective ducts or proximal tubule segments through the expansion of single clones^14^.

We previously showed that the tARPCs express TLR2 and that this receptor is able to influence the progenitor phenotype^11^. Here we demonstrate that tARPCs can sense damage through the TLR2 and can promote regeneration only if the receptor is expressed and functioning. In fact, when tARPCs were co-cultured with non-damaged RPTECs or when the TLR2 was neutralized, cell proliferation increase did not occur.

The mechanism by which renal progenitors induce the recovery of damaged cells is not only through the stimulation of damaged-cell proliferation but also through the arrest of the apoptotic process. In fact, tARPCs can inhibit the expression of the cleaved-caspase 3, an important marker of the cell’s entry point into the apoptotic signaling pathway^27^. The molecules that were involved in the ARPC repair are released by the cells after TLR2 damage sensing and they can act exactly on these biological processes. Both Inhb-A and DCN belong to the TGFβ pathway and are involved in apoptosis and proliferation processes^20–24^.

Inhibin-βA chains can form an homodimer called Activin. Inhibins and activins are two closely related protein complexes that have almost directly opposite biological effects. Inhb-A is involved in wounded skin repair and plays a role in cell proliferation, differentiation and apoptosis^28–31^. Moreover, Inhb-A also regulates the morphogenesis of branching organs such as prostate, lung, and especially kidney^32^.

Inhibins/activins and other members of the TGF-β superfamily exert their biological effects by interacting with transmembrane receptors called ALKs (activin receptor-like kinases), including ALK 1–7^21, 33^. Interestingly, these receptors bind also bone morphogenetic proteins (BMPs) that play a fundamental role in stem cell biology, regulating morphogenesis, differentiation of embryonic stem cells^34^ and stem cell proliferation^35^. Besides, BMPs are re-expressed in the adult kidney following renal injury^36^, particularly in regenerating proximal tubules^37^. These common receptors are mediators of the interaction of inhibins/activins with BMPs^38, 39^, that we previously found overexpressed in ARPCs after acute kidney injury (in particular the BMP-2 together with ALK-2, ALK-3 and ALK-6 receptors)^17^.

DCN, a small leucine-rich proteoglycan, is a potent antagonist of TGF-β signalling and can antagonize the response of nephron progenitor cells to BMP-7, that has been previously demonstrated to be essential for nephron progenitor cell differentiation^40^. Moreover, DCN can also be activated through WNT signaling^41^. These data link the DCN with very recent data from *in vivo* genetic lineage tracing and clonal analysis showing that renal progenitors are activated by WNT signals and can regenerate new tubule segments in damaged kidney^14^.

Moreover, we assembled a biocompatible polysaccharide core-shell system^42, 43^ able to include Inhb-A and DCN in its alginate hydrogel core. Results obtained from the experiments with ARPC MVs and from SVs indicate that, on one hand, DCN alone is not sufficient to induce damaged-RPTEC proliferation but it contributes in some way to the repair mechanisms, plausibly regulating the process. DCN may be secreted at levels not sufficient to induce *per se* the inhibiting function and therefore may only have a synergic effect in this process. Furthermore, additional *in vivo* studies are needed to establish whether Inhb-A alone is sufficient to support RPTECs regeneration or if other regenerative factors are needed.

Unexpectedly, *in vitro* administration of SVs carrying Inhb-A and DCN gave the same functional effect as the two proteins free in the medium. SVs could be very useful *in vivo* since they can be decorated with proteins that can be recognized by specific cells (i.e. RPTECs) and can ensure a specific delivery of the regenerative molecules.

In conclusion, we have shown that tARPCs can protect RPTECs from cisplatin toxicity by both avoiding necrosis/apoptosis and enhancing proliferation of survived cells. These repair processes occur after TLR2 activation and are mediated by the secretion of Inhb-A and DCN directly as proteins or as vehicled mRNAs in microvesicles. Our findings may be useful for the development of new therapeutic strategies to improve renal repair or by enhancing TLR2 signaling or directly shuttling reparative molecules in SVs to specific renal compartments.

## Methods

### Patients

All patients at the time of radical nephrectomy gave signed informed consent for the use of their tissue for research purposes. Portions of normal-appearing cortex were isolated surgically and examined histologically to exclude the presence of carcinoma. Concerning patients undergoing kidney transplant, according to our clinical practice protocol, a wedge biopsy before transplantation was performed on all cadaveric donor kidneys. In patients with DGF, we performed a second graft biopsy at 7 days after transplantation. The presence of DGF was defined as the need for dialysis in the first week after transplantation. Biopsies of apparently normal tissue fragments obtained from kidneys removed for renal cell carcinoma were used as a control group. All the collected renal biopsy specimens were fixed in 4% formaldehyde, paraffin-embedded, and processed for routine histologic staining and for immunofluorescence. All pre-transplant biopsies were scored by two independent pathologists. The study was carried out according to the principles of the Declaration of Helsinki and was approved by the Interregional Ethics Committee of the University Polyclinic of Bari. Every patient signed an informed consent form agreeing to participate in the study.

### Co-culture experiments

We isolated and characterized human ARPCs as previously described^11, 15–17^. Human RPTECs were purchased from ATCC- LGC (ATCC-LGC Standards S.r.l., Sesto San Giovanni, Milan, Italy) and Lonza (Lonza, Basel, Switzerland), respectively. RPTECs and HK2 were maintained in the recommended medium, D-MEM F12 (Sigma Aldrich, St. Louis, MO) containing 10% FBS, 100 U/ml penicillin, and 100 ng/ml streptomycin and REGM (Lonza), respectively. RPTEC medium was serum-free and all RPTEC and ARPC co-culture were performed in RPTEC medium. For *in vitro* experiments, RPTECs were plated at a density of at 10000 cells/cm^2^, and 48 h later they were incubated in medium alone or in presence of 2.5 mmol/l cisplatin for 6 h. For co-culture experiments, ARPCs were seeded on top of 0.4-mm-thick polycarbonate inserts (Costar Corning, Life Sciences, Acton, MA) at 8000 cells/cm^2^ in RPTEC medium, and were used for co-cultures after 2 days of quiescence (serum-free medium). RPTEC medium was also serum free. The ratio between the number of ARPCs and that of RPTECs was about 1/3.

After cisplatin removal, cells were plated in flat-bottomed 96-well plates (Costar Corning Inc., Life Sciences, Acton, MA, USA) and cell proliferation was measured by bromodeoxyuridine (BrdU) incorporation during last 6 h of a 2-days culture by a colorimetric immunoassay, according to the manufacturer’s guidelines (Roche Diagnostics, Mannheim, Germany). Untreated cells were used as controls. BrdU incorporated into the DNA was detected using an anti-BrdU peroxidase-conjugated antibody and visualized with a soluble chromogenic substrate. Values were acquired as absorbance at 450 nm - absorbance at 690 nm. The proliferation was calculated as the ratio between the BrdU absorbance in the wells of the various conditions of the damage model and the control wells containing not-damaged RPTECs. Necrosis was induced by treatment for 12 h with 6 mM deoxyglucose and 10 mM sodium azide^19^.

Blocking experiments for TLR2 and Inhb-A were performed using the TLR2 (Biolegend, San Diego, CA) and Inhb-A (Novus Biologicals, Littleton, CO) antibodies. The TLR2 antibody (2 ng/ml) was added directly on ARPCs plated in transwell for 1 hour and several washes were performed to remove unbound antibody before adding the transwell to cisplatin-damaged RPTECs. Inhb-A antibody was added (1 ng/ml) both in transwell and in RPTEC wells.

In specific experiments, supernatants were treated with 1 U/ml RNase (Ambion, Austin, TX) for 1 h at 37 °C; the reaction was stopped by the addition of 10 U/ml RNase inhibitor (Ambion). The effectiveness of RNase treatment was evaluated after RNA extraction by Agilent Bioanalyzer (Agilent Technologies, CA, USA) analysis of total extracted RNA.

MVs were isolated from supernatants of RPTECs and ARPCs in the different conditions of the damage model by serial centrifugation, removing cells (300 g, 10 min) and non-cellular debris (17000 g, 20 min). The supernatant was, then, subjected to 2 serial ultracentrifugation at 118,000 g for 70 min.

Inhb-A levels were measured by Inhb-A enzyme-linked immunosorbent assay (ELISA) kits (Quantikits, R&D Systems, Minneapolis, MN, USA), according to the manufacturer’s instructions.

### Wound healing assay

RPTECs were plated at 1 × 10^5^ in 6-wells plate. A scratch was performed on tubular cell monolayers to simulate a physical damage. Three independent wounds (~20–25 mm) per dish were established with a sterile pipette tip. Subsequently, tubular cells were incubated alone or in co-culture with ARPCs on transwells at the density of 80 × 10^3^. RPTECs scratched and cultured without tARPCs were used as control. Images were captured at phase contrast microscopy at time intervals of 0, 24 and 48 hours to detect the gradual repair of the gap. The scratch assay was analyzed by the MRI Wound Healing Tool of the ImageJ software. After image analysis, results were expressed as a ratio between area of scratches at T48 and at T24 respect to the scratch area at T0.

### Flow-cytometry analysis

For surface staining, cells were resuspended in flow cytometry (FACS) buffer (phosphate-buffered saline, pH 7.2, 0.2% bovine serum albumin, and 0.02% sodium azide) and incubated with FCR blocking reagent (Miltenyi Biotec) for 10 minutes at room temperature. After blocking incubation, surface markers were added for 15 minutes at 4 °C. Then cells were washed with the FACS buffer and were resuspended in each tube with 500 μl of FACS buffer. Intracellular staining for cleaved-caspase 3 was preceded by fixation and permeabilization with IntraPrep kit (Instrumentation Laboratory) and incubation for unconjugated primary antibody (rabbit IgG cleaved caspase-3 (Asp175) mAb, R&D Systems) 25 min at 4 °C. Cells were then washed and labeled with secondary antibody AlexaFluor 488 (Molecular Probes) for 25 min at 4 °C. Finally, cells were washed twice and resuspended in FACS buffer for acquisition. Data were obtained by using a FC500 (Beckman Coulter) flow cytometer and analyzed with Kaluza software. Three independent experiments were performed. The area of positivity was determined by using an isotype-matched mAb, and in total, 10^4^ events for each sample were acquired.

### Immunostaining and confocal microscopy

The cells were blocked for 1 h (BSA in PBS, pH 7.4) and then incubated with primary antibodies overnight at 4 °C or for 2 h at room temperature, respectively. The immune complexes were identified with the respective specific secondary antibodies for 1 h at room temperature. Paraffin-embedded human renal biopsy sections were deparaffinized, rehydrated, treated for antigen retrieval and incubated in blocking solution prior to the incubation with primary antibodies at room temperature (RT) for 1 hour or at 4 °C overnight according to different antibodies.

The following primary antibodies were used: mouse anti-human TLR2 mAb (HyCult Technologies, Plymouth Meeting, PA), rabbit anti-human inhibin A pAb (Novus Biologicals,), mouse anti-human CD133 mAb (Miltenyi Biotec), rabbit IgG cleaved caspase-3 (Asp175) mAb (R&D Systems). The following secondary antibodies were used: Alexa Fluor 555 goat anti-mouse IgG, Alexa Fluor 488 goat anti-rabbit IgG, and Alexa Fluor 488 goat anti-mouse IgG1 (all from Molecular Probes). All cells and sections were counterstained with To-pro-3 (Molecular Probes, Eugene, OR, USA) and mounted in Fluoromount (Leica, Wetzlar, Germany). Negative controls were prepared with irrelevant antibody. The stained cells were viewed under the Leica TCS SP2 (Leica, Wetzlar, Germany) confocal laser-scanning microscope using ×40 and ×63 objective lenses.

### Depletion of TLR2^+^ ARPCs

tARPCs or gARPCs were detached by trypsin/EDTA digestion, counted and resuspended with recommended medium (PBS pH 7.4 with 2% FBS and 1 mM EDTA) in polystyrene round-bottom tube. Then, cells were incubated with TLR2 positive selection cocktail (EasySep, Stemcell, Vancouver, Canada) at concentration of 100 µl/ml cells, at room temperature for 15 min. EasySep Magnetic Nanoparticles were added to cell suspension at concentration of 50 µl/ml cells and incubated at room temperature for 10 min. Cells were resuspended with recommended medium and cell tube was placed into the EasySep Magnet. After 5 min the magnet within cell tube was inverted and leaved for 2–3 sec, pouring off the supernatant fraction. The TLR2 positive cells remained inside the tube held by magnetic field of EasySep Magnet. Resuspension with recommended medium and Magnet steps were repeated for 3 times. Finally, cell tube was removed from magnet, both TLR2 positive and TLR2 negative cells were resuspended in EGM-MV with 20% FCS and incubated at 37 °C with 5.0% CO2.

### Silencing

For TLR2 silencing, siRNA was purchased from Qiagen (Valencia, CA, USA) in 3 formulations. A non-silencing siRNA sequence, shown by Basic Local Alignment Search Tool (BLAST) search to not share sequence homology with any known human mRNA and tagged with Alexa Fluor 488 (AllStars Negative Control siRNA; Qiagen), was used to determine uptake transfection efficiency. For *in vitro* delivery, siRNA (50 nM) was incubated with 5 µl TKO reagent (Mirus, USA) for 15 min at room temperature and added to cells in culture at 80% confluence in Transwell. All procedures were performed according to the manufacturer’s instructions. Silencing was confirmed by real-time PCR. Functional effects on co-cultures were observed when inhibition of TLR2 expression was 40% and higher. The TLR2 silencing efficiency was about 75%.

### Transmission Electronic Microscopy

Transmission electron microscopy was performed on cultured ARPCs releasing MVs through supernatants. ARPCs-derived supernatants were fixed in 2% GTA for 1 hour, dehydrated in alcohol, osmium tetraoxide-postfixed for 1 hour and embedded in epoxy resin according to standard procedures. Ultrathin sections were stained with uranyl acetate and lead citrate and were examined with a ZEISS 910 electron microscope. For scanning electron microscopy images were obtained via secondary electron at a working distance of 15 to 25 mm and an accelerating voltage of 20 to 25 kV. Image acquisitions were performed with magnification of ×12500, ×16000 and ×25000.

### RNA Extraction and Real-Time PCR

Total RNA extraction was performed by means of miRNeasy Mini kit (Qiagen) according to the manufacturer’s protocol. DNase treatment was carried out to remove any contaminating DNA (RNase-Free DNase Set, Qiagen). The RNA concentration was determined with NanoDrop Spectrophotometer (Nanodrop Technologies, Wilmington, DE, USA). Total RNA was reverse transcribed with QuantiTect Reverse Transcription Kit (Qiagen) following the manufacturer’s instructions. Quantitative RT-PCR amplification reactions were performed in triplicate in 25 μl final volumes using SYBR Green chemistry on an iCycler. Quantitative RT-PCR was performed using the QuantiFast SYBR Green PCR mix (Qiagen). Genes were amplified according to the manufacturer’s directions. The β-actin gene amplification was used as a reference standard to normalize the target signal. Primers used for the RT-PCR are: INHB-A Fw, GGTACCCAAGGCGGCGCTTC; INHB-A Rv, TGGCTGTTCCTGACTCGGCA; DCN Fw, TGGGTGTCAGCCGGATTGTGTT; DCN Rv, CAACCAGGGAACCTTTTAATCCGGG.

### SV preparation

Alginic sodium salt (low viscosity) (AL) and chitosan (medium molecular weight) (CS) were exploited in order to fabricate a biocompatible core-shell polymer-based synthetic vesicles for efficacious combination of Inhb-A and DCN, under mild gelation conditions.

Inhb-A- and DCN-SV were synthesized by a two steps method: i) ionotropic pre-gelation of AL nanogel core with CaCl2 by hydrodynamic flow focusing on a cross-junction microfluidic platform^44^, followed by ii) bulk CS polyelectrolyte complexation, in order to obtain SV with an external CS shell. The microfluidic device was appositely fabricated by using soft-litography technique^45^ in order to control and optimize the vesicle assembly process, in terms of polymer and protein amount, as well as vesicle size distribution. CaCl2 aqueous solution (0,67 mg/ml) was adopted as AL (0,6 mg/ml) reticulating agent. For protein encapsulation, experimentally selected amount of Inhb-A- and DCN- (see next paragraph) were dissolved in CaCl2 solution immediately before injection in the device microchannel. Inhb-A- and DCN- loaded nanogel core, as well as blank core were collected in the supernatant fraction by sample centrifugation at 13000 rpm. After nanogel core synthesis and stabilization, CS shell was obtained by bulk mixing the AL nanogel core with the oppositely charged CS (1.2 mg/ml in acetic acid 1% v/v) under magnetic stirring for 2 h. Final submicrometric SV purification and concentration (mean diameter, 500 nm) was performed on the 100 kDa cut off - Vivaspin Ultra centrifuge tube (Sartorius Goettingen, Germany) at 4000 rpm.

### Exogenous cell stimulation

*In vitro* stimulation by Inhb-A or DCN on cisplatin-damaged RPTECs was performed directly free in the medium at serial concentrations of 1, 3 and 5 ng/ul to identify effective concentration. Then, the 5 ng/ul concentration was used for the experiments. Cell proliferation was measured by BrdU assays.

### Statistical analyses

We analyzed data with statistical software GraphPad Prism (GraphPad, San Diego, CA, USA). All results are expressed as mean ± s.e.m. All values are expressed as the mean of data obtained from at least three independent experiments. Two-tailed Student’s t-test has been used to assess differences in biological features between two mean values.

## Electronic supplementary material

Supplementary Information
