# Supplementary material for: Inhibin-A and Decorin Secreted by Human Adult Renal Stem/Progenitor Cells Through the TLR2 Engagement Induce Renal Tubular Cell Regeneration
Source: Sci Rep. 2017 Aug 15;7:8225. doi: 10.1038/s41598-017-08474-0 (PMC5557965; doi:10.1038/s41598-017-08474-0)
Supplement: Supplementary file 1 — Supplementary Information [file 41598_2017_8474_MOESM1_ESM.pdf]

Inhibin-A and Decorin Secreted by Human Adult Renal Stem/Progenitor Cells Through the TLR2 Engagement Induce Renal Tubular Cell Regeneration

AUTHORS

Fabio Sallustio, Claudia Curci, Alessandra Aloisi, Chiara Cristina Toma, Elisabetta Marulli, Grazia Serino, Sharon Natasha Cox, Giuseppe De Palma, Alessandra Stasi, Chiara Divella, Rosaria Rinaldi, Francesco Paolo Schena.

Supplementary Information

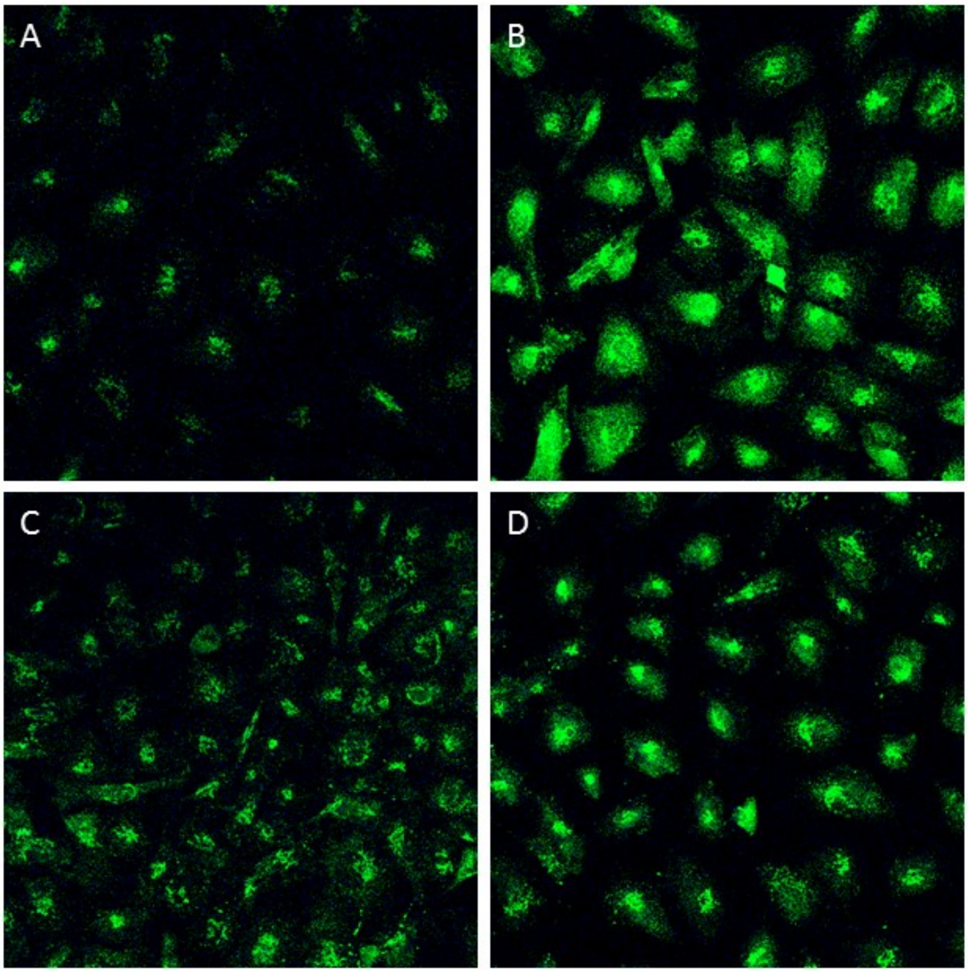

Figure S1. tARPCs can abrogate cisplatin-induced apoptosis of RPTECs.

Immunofluorescence stainings show that the cleaved-caspase 3 expression on cisplatin-damaged RPTECs significantly increased (B) compared to untreated cells (A) after one day of cell culture. Instead, when the RPTECs were co-cultured with tARPCs, the cleaved-caspase 3 was less expressed (C). Panel D represents a positive control of cleaved-caspase 3 reaction on RPTECs treated with H_2O_2. Magnification 630x.

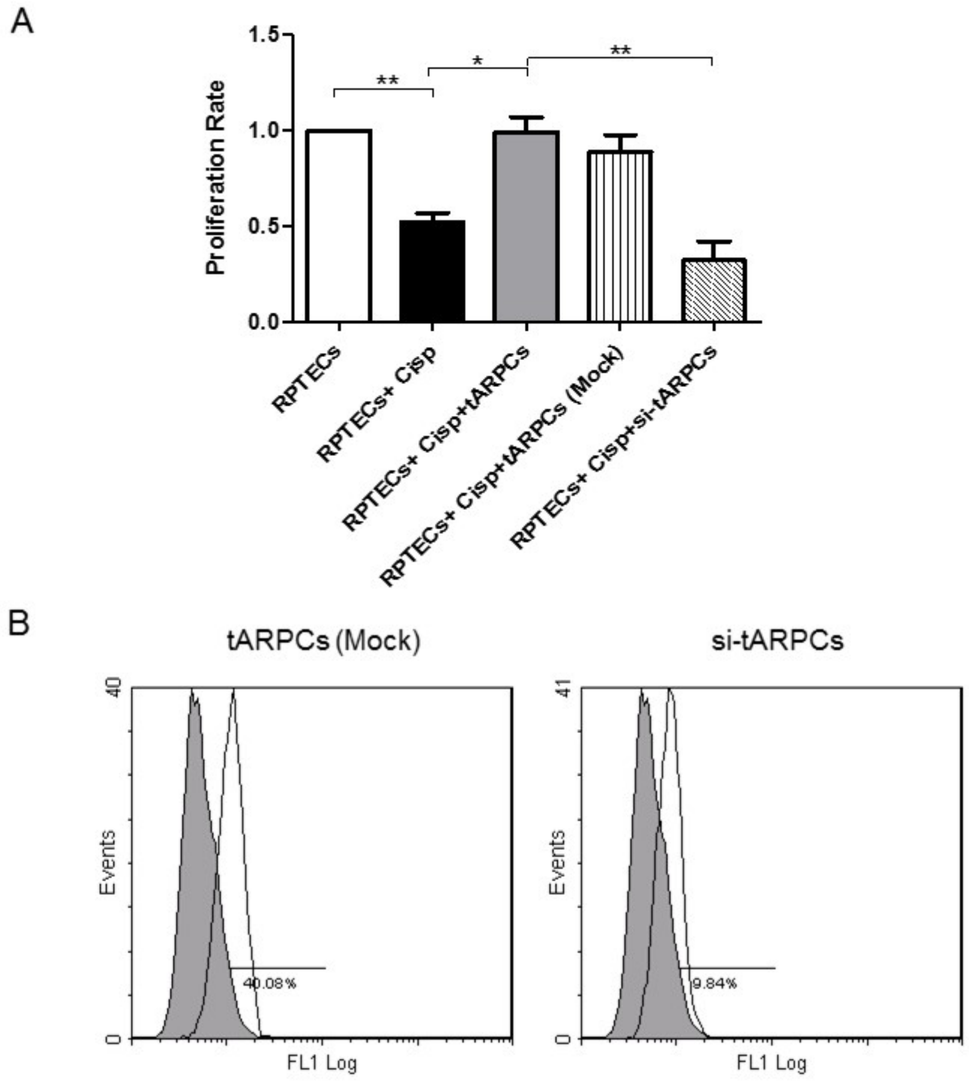

Figure S2. The TLR2 is responsible for tubular cell regeneration.

A) A significant inhibition of the recovery process was found when tARPCs were silenced with small interfering RNA for the TLR2 and co-cultured with cisplatin-damaged RPTECs compared to non-silenced tARPCs. The abrogation of the functional effect was observed when TLR2 expression inhibition was approximately 40% or higher. RPTECs without cisplatin were used as control. The mock-transfection control was obtained when cells underwent the cell transfection procedure without adding siRNA. *= ≤ 0.05, **= ≤ 0.005. B) Efficiency of TLR2 silencing was evaluated by FACS analysis. ARPCs expressing the TLR2 decreased from 40% to 10% after silencing, showing that the efficiency was about 75%.
